# Biophysics-Guided Lead Discovery of HBV Capsid Assembly Modifiers

**DOI:** 10.1021/acsinfecdis.3c00479

**Published:** 2024-04-02

**Authors:** Zixing Fan, Anna Pavlova, Matthew C. Jenkins, Leda Bassit, Mohammad Salman, Diane L. Lynch, Dharmeshkumar Patel, Maksym Korablyov, M. G. Finn, Raymond F. Schinazi, James C. Gumbart

**Affiliations:** † Interdisciplinary Bioengineering Graduate Program, Georgia Institute of Technology, Atlanta, Georgia 30332, United States; ‡ School of Physics, Georgia Institute of Technology, Atlanta, Georgia 30332, United States; § School of Chemistry & Biochemistry, Georgia Institute of Technology, Atlanta, Georgia 30332, United States; ∥ Center for ViroScience and Cure, Laboratory of Biochemical Pharmacology, Department of Pediatrics, Emory University School of Medicine and Children’s Healthcare of Atlanta, Atlanta, Georgia 30322, United States; ⊥ MIT Media Lab, Massachusetts Institute of Technology, Boston, Massachusetts 02139, United States; ¶ School of Chemistry & Biochemistry and School of Biological Sciences, Georgia Institute of Technology, Atlanta, Georgia 30332, United States

**Keywords:** HBV, molecular dynamics, docking, CAM

## Abstract

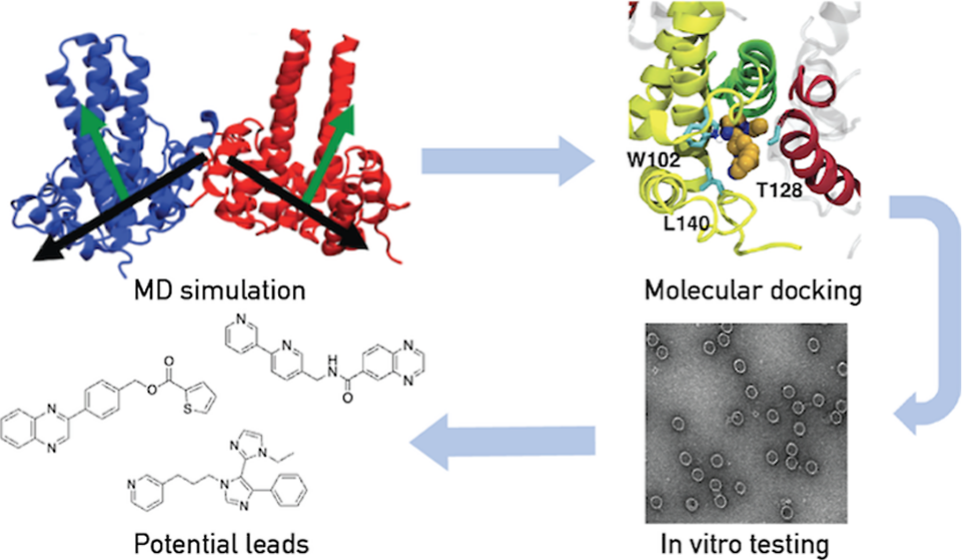

Hepatitis B virus (HBV) is the leading cause of chronic liver pathologies worldwide. HBV nucleocapsid, a key structural component, is formed through the self-assembly of the capsid protein units. Therefore, interfering with the self-assembly process is a promising approach for the development of novel antiviral agents. Applied to HBV, this approach has led to several classes of capsid assembly modulators (CAMs). Here, we report structurally novel CAMs with moderate activity and low toxicity, discovered through a biophysics-guided approach combining docking, molecular dynamics simulations, and a series of assays with a particular emphasis on biophysical experiments. Several of the identified compounds induce the formation of aberrant capsids and inhibit HBV DNA replication in vitro, suggesting that they possess modest capsid assembly modulation effects. The synergistic computational and experimental approaches provided key insights that facilitated the identification of compounds with promising activities. The discovery of preclinical CAMs presents opportunities for subsequent optimization efforts, thereby opening new avenues for HBV inhibition.

Chronic hepatitis B virus (HBV) infection affects around 250 million people worldwide, causing approximately 800,000 deaths each year due to liver complications.^1^ Although a vaccine exists, once infected, the persistent presence of covalently closed circular DNA (cccDNA) in the nuclei results in a chronic infection. This episomal cccDNA is not eliminated by presently approved therapies,^2−4^ requiring long-term therapeutic treatment, thus motivating the continued development of novel antiviral treatments. A promising orthogonal approach for eliminating the infection, perhaps in combination with antiviral or immune therapies, is to target the HBV nucleocapsid assembly.^5−14^ The HBV capsid comprises 240 copies of the core protein (Cp) forming an icosahedral protein shell, while the Cp primarily exists as a homodimer in solution under nonassembling (low ionic strength) conditions.^2^ The N-terminal domain of Cp (Cp149) is sufficient for forming regular capsids,^15^ while the arginine-rich C-terminal region (residues 150–183) is needed for pregenomic RNA (pgRNA) encapsidation among other functions.^16,17^ The Cp149 dimer consists of two domains: the dimerization interface consisting of helices α3 and α4 (Figure 1A) and the assembly interface responsible for forming interdimer contacts (helices α1, α2, and α5, Figure 1B).^18,19^

**Figure 1:**
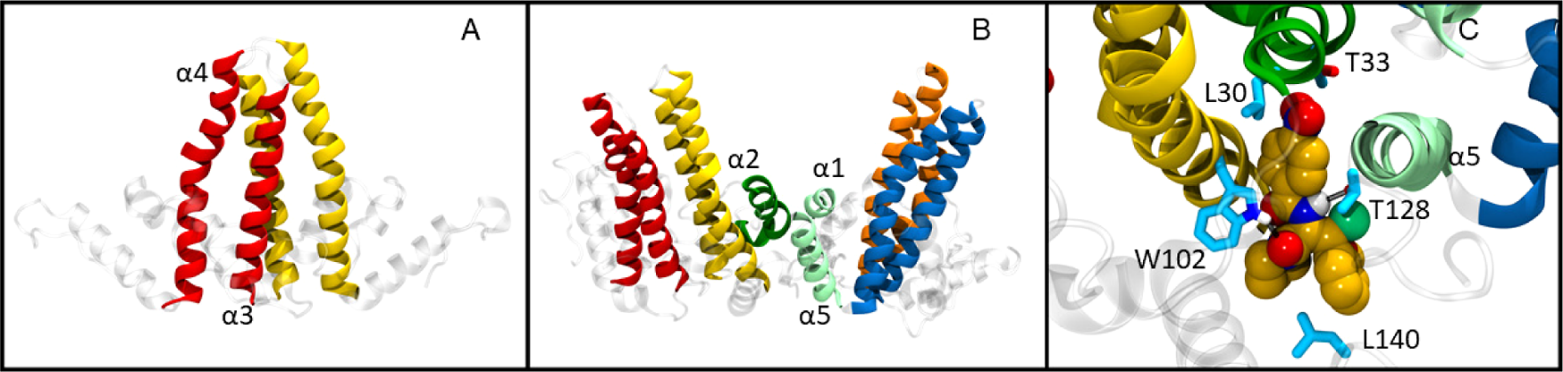
Structure of Cp149. (A) Dimerization interface, with the α3 and α4 helices rendered as red (monomer 1) or yellow (monomer 2) ribbons. (B) Assembly interface, with α2, α1, and α5 rendered in dark/light green. (C) Binding pocket with AT130 (PDB 4G93) bound and several of the ligand-binding residues. Highlighted carbon, oxygen, nitrogen, hydrogen, and chlorine atoms are rendered in gold, red, blue, white and light green spheres, respectively.

Previous studies proposed that the Cp149 dimers trigger capsid assembly by adopting an energetically unfavorable “assembly active” conformation, which in turn leads to assembly nucleation.^20−23^ It was also concluded that the assembly is nucleated by the formation of a hexamer, a triangular trimer of dimers, which is the rate-limiting step and is followed by the successive addition of the dimers or other small intermediates, e.g., tetramers or hexamers, until the complete nucleocapsid is formed.^20−22^ Several factors, such as ions,^20−22^ mutations,^24−26^ and some small molecules named capsid assembly modulators (CAMs)^5−10^ alter the kinetics and/or thermodynamics of HBV capsid assembly, potentially preventing the formation of normal capsids and, in some cases, localizing the capsid in the cytoplasm.^27^

The CAMs are small molecules that affect capsid assembly by interacting with the capsid proteins.^5−10,12,13^ A number of structurally and mechanistically distinct CAMs targeting HBV have been discovered. Different assembly effects, such as acceleration or misdirection, have been achieved in a ligand-dependent fashion.^5−13^ For example, heteroaryldihydropyrimidines (HAPs) cause the formation of nonspherical structures, e.g., tubes or sheets,^5,6^ whereas phenylpropenamides (PPAs)^7,8^ and sulfamoyl benzamides (SBAs)^9−11^ induce the formation of normal spherical capsids, albeit lacking the viral pgRNA. Although both PPAs and SBAs cause the formation of empty capsids, it has been shown that some PPAs, e.g., AT130, also increase the assembly rate of Cp149;^8^ however, no changes in the assembly rates were observed for Cp149 assembly with and without SBAs, suggesting that SBAs and PPAs alter the capsid assembly differently.^9,10,28^ CAMs based on a glyoxamidopyrrolo backbone (GLP-26) have also been reported.^27,29^ These CAMs showed the formation of spherical, misshapen particles, distinct from the structures observed for other CAMs.^27^ Moreover, one of them, GLP-26, displays robust low-nanomolar activity in vitro and demonstrated reduction of HBV DNA and other HBV markers in a humanized HBV mouse model,^27,29^ while one of its related derivatives (ALG-184) is in phase 1b clinical development.^14^ Novel chemotypes such as phthalazinones^30^ and pyrazoles^14^ have also been reported as CAMs that effectively inhibit HBV DNA replication.

Almost all known CAMs bind in the pocket at the assembly interface as shown by crystal and cryogenic electron microscopy (cryo-EM) structures (Figure 1C).^10,31−33^ These studies revealed several hydrophobic contacts between the CAM and protein residues and slightly altered dimer–dimer orientations,^31,32^ resulting in altered tertiary and/or quaternary structures. Continued advances in solid-state nuclear magnetic resonance (NMR)^34^ have enabled the investigation of conformational changes of dimers during capsid assembly, where it was shown that the actions induced by different classes of CAMs are distinguishable.^35^ Both viral capsids and transient assembly intermediates with and without bound CAMs have been studied with molecular dynamics (MD) simulations, providing insights into their mechanisms of action. Altered capsid dynamics upon CAM binding have been demonstrated.^26,36^ In our previous work,^37^ we performed MD simulations of Cp149 tetramers and hexamers with several known CAMs and concluded that different structural CAM classes induce distinct structural changes in the protein, with flatter structures observed for assembly misdirecting HAP compounds and more curved structures for agents that induce nonreplicative icosahedral capsid formation.

Several CAMs are already in clinical testing,^14,27,38^ showing their promise in antiviral treatment and making assembly misdirection worthy of further exploration. Development of new CAMs has usually involved high-throughput screening (HTS) of large libraries of chemical compounds to identify potential leads. Virtual screening, employing computational methods to identify potential lead compounds and enabling rational design,^39^ is a potentially more efficient method for drug discovery and has led to the discovery of some novel CAM hits.^40^

In this work, we continue our emphasis on generating a unified biophysical approach to a novel hit-to-lead generation appropriate for early-stage drug development.^37^ In fact, experimental biophysical methods have had a successful impact on this process and, along with computational methods, are currently a central component of drug candidate characterization. Here, we combine MD, docking, and in vitro experiments to develop novel CAMs targeting HBV and elucidate their mechanisms of action. We find that our compounds induce changes to interdimer conformations in MD simulations in a manner similar to that by HAP agents, and biophysical and in vitro experiments validate the effects on assembly for some of them. By determining their binding properties and mechanisms of action early in the drug development cycle, we can improve the efficiency of the hit-to-lead generation campaigns for our early preclinical candidates. Thus, these compounds have potential for further development as antiviral treatments for chronic HBV infection.

## Results

### Discovery of Novel CAMs by Virtual Screening

In a previous computational study, we found that the calculated drug-induced differences in the interdimer orientation of early assembly intermediates of HBV capsid (such as Cp149 tetramers or dimers of dimers) were predictive of the overall effect of the CAM molecule.^37^ The tetramer is also the asymmetric subunit of T4 HBV capsid, and it is the smallest intermediate for which interdimer structural changes can be observed.

To further explore the hypothesis that structures of early assembly intermediates could be used for the development of novel CAMs, we performed principal component analysis (PCA) on our two previously reported simulations of the apo Cp149 tetramer, with the top three components representing interdimer motions. Three structures from these simulations (Tetra1, Tetra2, and Tetra3) were selected for docking due to both their large pocket volume and differences from capsid structures in principal component space (Figure S1). Structural alignment showed that the most distinct regions for the three selected structures are the α5 helix and the C-terminus of the C chain (Figure S11). Our previous MD simulations showed that the pocket volume in the apo tetramers is smaller in comparison to that of tetramers with bound compounds. Therefore, we aimed to select the apo structures with a larger-than-average pocket size. Two databases were selected for the initial docking: the DivV set from NCI^41^ and ZINC0.9, which consist of all compounds with a Tanimoto similarity coefficient of 0.9 or lower in the ZINC database.^42^ Both databases were docked to all three selected protein structures, and the top 100 ranked compounds from the combined results of both databases for each structure were considered for testing (see the Methods section for details on compound filtering). In total, 29 compounds were selected for experimental testing: 11 from the DivV set and 18 from the ZINC0.9 database (see Figures S2 and S3 for structures and numbering).

The compounds were tested in HepAD38 cells over 7 days to evaluate the HBV DNA inhibitory effects, and they were also evaluated for cytotoxicity in four cell lines including human peripheral blood mononuclear (PBM), T lymphoblast CEM-CCRF (herein referred to as CEM cells), African green monkey kidney (Vero), or human liver carcinoma (HepG2) cells. Table S1 shows the tested compounds’ activity, toxicity, and docking score. Among all of the tested compounds, GT-5 was the only one showing moderate antiviral activities as well as low cytotoxicity (CC_50_ > 100 μM) in all cell lines and thus was selected as the hit to be optimized further. GT-9 showed similar performance but was not developed further because of its much more hydrophobic nature, placing it at a disadvantage for elaboration into drug-like structures.

### Potency Optimization of CAMs

**Figure 2:**
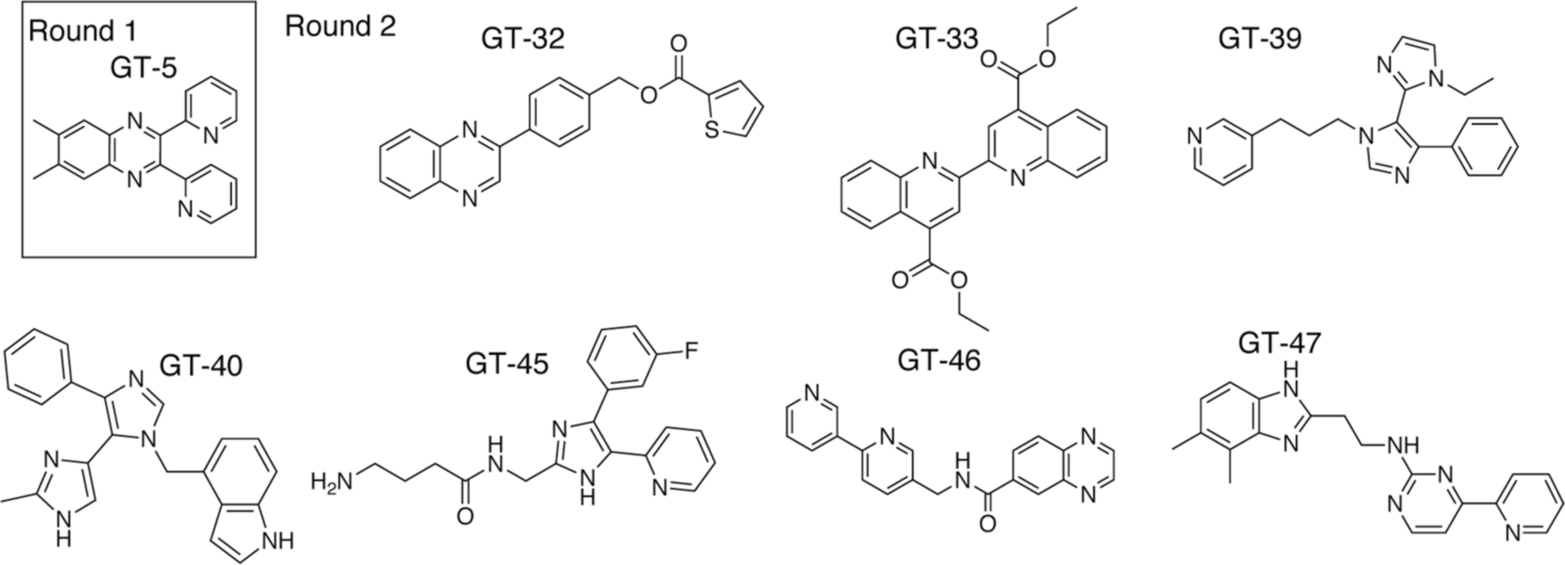
Structures of our compounds that showed moderate activity against HBV.

Starting from GT-5, we performed a second round of docking using structurally similar compounds and the protein structure Tetra2, for which GT-5 scored as one of the top compounds. The NCI and Molport databases were searched for compounds with a similarity of at least 0.7 (Tanimoto coefficient),^41^ finding ∼2000 compounds. These compounds were docked to the Tetra2 structure in this round. Compounds with GlideXP docking scores lower than −8.0 kcal/mol were added to the list of potential new leads and filtered based on their properties as described in the Methods section. Several compounds were also removed from the list due to a bad overlap with the docking pose of GT-5 based on visual inspection. In total, 20 new compounds were selected for experimental testing (see Figures S4 and S5 and Table S2). The candidate compounds were evaluated for their ability to inhibit HBV DNA replication in duplicate. A number of compounds exhibited modest inhibition in HBV DNA replication at a concentration of 10 μM in HepAD38 cells (see Table S2), suggesting that the GT-5 motif was a promising starting point. However, discrepancies in the activities of these compounds were observed between the two trials. The limitations inherent to in vitro assays, as previously elucidated within the context of Ebola virus research,^43^ underscore the complexities encountered in the early phases of drug discovery. It is pertinent to acknowledge that our study remains in the nascent stages of drug development, and the efficacy of the compounds under study is yet to reach a level of marked potency. Given the potential for significant variability in cellular-based assays, it is imperative that any compounds demonstrating the inhibition of HBV DNA replication in at least one of the preliminary trials be subjected to further scrutiny. This subsequent analysis will involve assessing their capacity for misdirection via direct biophysical assays, thereby ensuring a comprehensive evaluation of their potential for further development. Seven compounds (GT-32, GT-33, GT-39, GT-40, GT-45, GT-46, and GT-47), which showed more than ∼50% inhibition in at least one of the trials (Table 1), were selected for further analysis (Figure 2). These chosen compounds were subjected to additional tests for HBV DNA replication inhibition in two more trials, and the observed variations continued, as reported in Table S3. This led to the need for more in-depth biophysical assays. Five of these compounds did not exhibit relevant toxicity at the effective concentration, while two of them (GT-39 and GT-47) displayed low to moderate toxicity (16–18 μM) in at least one cell line tested.

**Table 1:** Experimental and Computational Data for Compounds Displaying Inhibition of HBV DNA Replication in At Least One Trial^a^

comp.	% HBV DNA Inh. at 10 μM (EC_50_ in μM)		docking	MTT cytotoxicity, CC_50_ (μM)			
	trial 1	trial 2	(kcal/mol)	PBM	CEM	Vero	HepG2
GT-5	24		–8.40	>100	>100	>100	>100
GT-32	48 (≥10)	<1 (>10)	–8.01	85	52	44	59
GT-33	60 (<10)	37 (>10)	–8.36	>100	>100	76	86
GT-39	57 (<10)	50 (10)	–9.96	>100	16	>100	>100
GT-40	55 (<10)	50 (10)	–9.52	>100	77	>100	>100
GT-45	50 (≥10)	49 (≥10)	–10.86	>100	>100	66	>100
GT-46	49 (≥10)	49 (≥10)	–9.81	>100	38	53	82
GT-47	49 (≥10)	49 (≥10)	–8.57	>100	>100	>100	18

a We display percentage inhibition (Inh.) of HBV DNA replication in HepAD38 cells at 10 μM compound concentration. The docking scores to the Tetra2 structure are shown. Finally, toxicity in four different cell lines is shown as CC_50_.

### MD Simulations of Active CAMs

**Figure 3:**
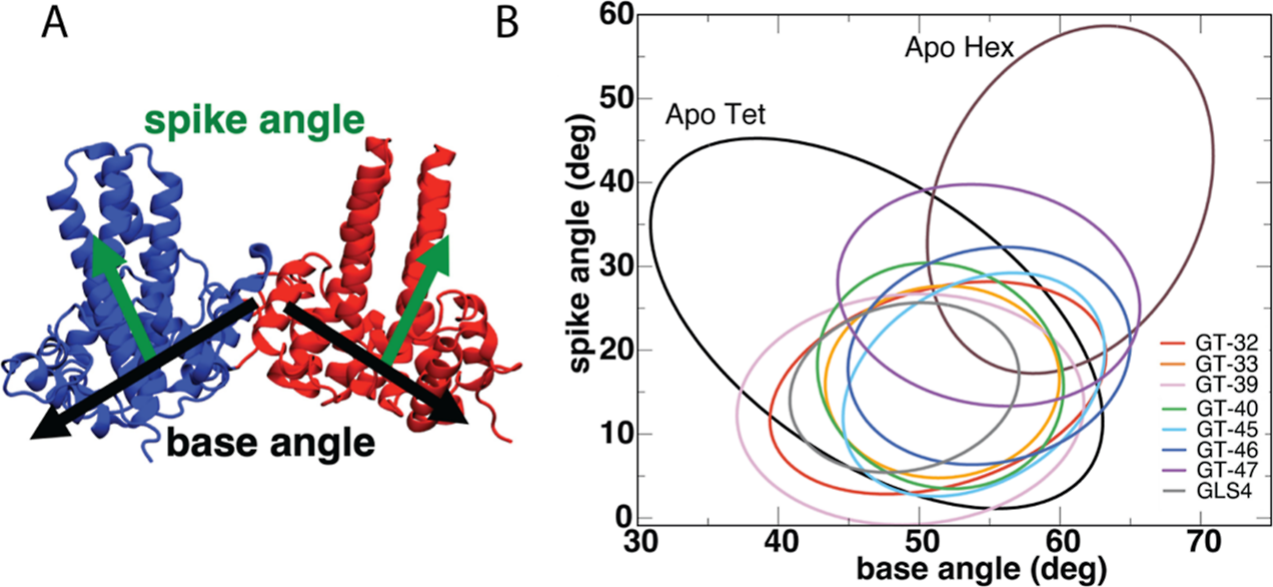
(A) Definitions of spike and base angles. (B) SDEs of the base and spike angles calculated from the MD simulations of tetramers with bound novel compounds. Results for the Apo tetramer and hexamer SDEs as well as GLS4-bound tetramer SDE from previous work^37^ are added for comparison.

MD simulations were used to investigate how our compounds alter the structure of early assembly intermediates and to determine their mechanism of action. We simulated the seven active compounds, GT-32, GT-33, GT-39, GT-40, GT-45, GT-46, and GT-47, bound to the Cp149 tetramers. As noted previously, the structural changes in early assembly intermediates upon CAM binding are well-described by base and spike angles^37^ (Figure 3A). The spike angle is calculated between the combined α3 and α4 helices of each dimer and describes the “bending” of the tetrameric unit. The base angle is calculated based on the positions of all α5 helices in each dimer and describes the “opening” and “closing” of the tetrameric unit. To illustrate the observed structural differences, the distributions of the spike and base angles were projected on a two-dimensional scatter plot, and standard deviations ellipses (SDEs) were calculated.^44^ Next, the fractional overlap area (FOA) between the compared systems was calculated as the overlap area of the two SDEs divided by their total area, and the results were compared to previous simulations of the Apo Cp149 tetramer and hexamer, as well as tetramer with bound GLS4.^37^ Our previous work showed that these parameters can distinguish between CAM mechanisms of action by the difference in location and shape of SDEs, as well as their overlaps with the Apo tetramer and hexamer.^37^

As shown in Figure 3B, the novel CAMs are predicted to induce significant changes in the conformations of Cp149 tetramers. Table S5 shows the ranges of the base and spike angles in all of the simulations, and Table S6 summarizes the FOAs between all pairs of simulations. GT-47 displayed the largest spike angles (13–40°) in comparison to those of the apo tetramer (1–45°) and other tested compounds (−1–32°). In addition, its FOAs with both apo tetramer and apo hexamer were significant (69 and 59%, respectively), while its overlap with GLS4 was not significant (39%). This profile is similar to those previously observed for GLP-based CAMs,^37^ which can induce the formation of misshapen capsids. Simulations with the remaining compounds (GT-32, GT-33, GT-39, GT-40, GT-45, and GT-46) displayed base and spike angles (37–63 and −1–32°, respectively) similar to those of the HAP class of misdirecting compounds such as GLS4 (41–57 and 5–26°, respectively). The ranges of the observed spike angles were lower for our compounds than for apo tetramer (1–45°), and their overlap with apo tetramer and GLS4 was highly significant (FOA >80 and >69%, respectively), while their FOA with apo hexamer was not significant (<39%).

The analysis of the base and spike angles suggests that these compounds might have misdirecting effects on capsid assembly. Among them, GT-39 has the smallest overlap with the apo hexamer (9%) and significant overlap with the apo tetramer (83%), which is comparable to GLS4 (8 and 100%, respectively), so it is expected to have misdirecting effects similar to those of the HAP compounds. Other compounds with the exception of GT-46 also showed high similarity to GLS4, whereas GT-46 data looked closer to what is expected of GLP-26 and related compounds.^37^ None of the simulated CAMs showed trends similar to those of the PPA class of accelerators, which have a small overlap with the apo tetramer and a significant overlap with the apo hexamer. Thus, these CAMs are expected to cause the formation of misshapen capsids rather than accelerate the assembly of normally shaped capsids. The observation of distorted conformations in MD, which is consistent with misdirection, was encouraging and motivated additional testing in biophysical assays.

### CAMs Directly Bind to Cp149

**Figure 4:**
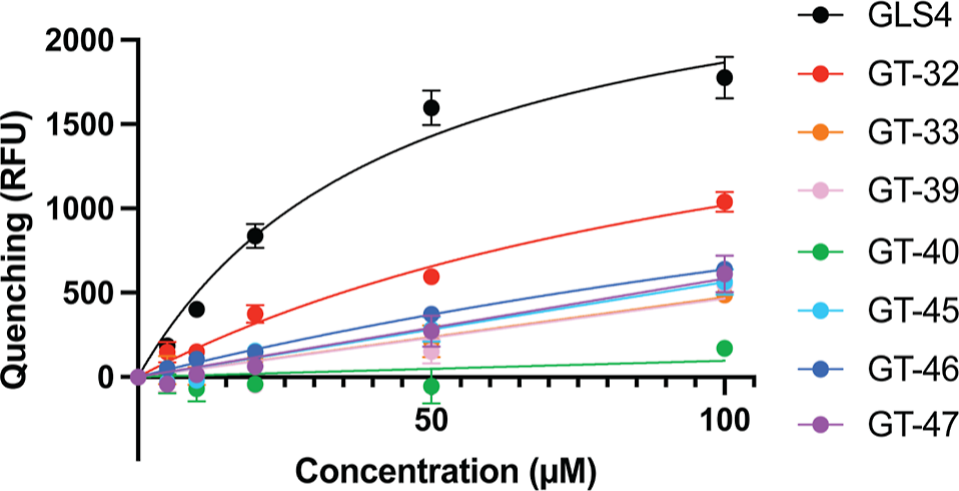
Tryptophan fluorescence titration curves for novel CAMs and GLS4. Dots and error bars represent the averages and standard deviations from three replicates, respectively. Curves for GLS4, GT-32, and GT-46 were fit to a single-site binding model in Graphpad Prism 9.^45^

The strength of binding of the selected CAMs to Cp149 was assessed by tryptophan fluorescence titration on recombinant Cp149 produced in *E. coli* BL21(DE3) cells and isolated as intact virus-like particles (VLPs). These purified capsids were disassembled at high pH in the presence of urea followed by dialysis to obtain clean Cp149 dimers. Bioanalyzer, dynamic light scattering (DLS), and native agarose gel electrophoresis (NAGE) analysis showed that the Cp149 dimers were successfully expressed and purified (Figures S6–S8, respectively). Seven of the CAMs selected above (along with GLS4 as a positive control) were titrated into a 20 μM solution of Cp149, and fluorescence quenching was measured after equilibration with the results shown in Figure 4 and Table S7. The dissociation constant (*K*_D_) of GLS4 was estimated to be 44 μM (31.8–63.0 μM), consistent with the previously reported value of 41 ± 13 μM.^27^ GT-32 and GT-46 showed moderate fluorescence quenching, which indicated direct binding to Cp149 and allowed the estimated *K*_D_ values to be determined as 124 μM (73.1–256.9 μM) and 263 μM (156.0–615.4 μM), respectively. No fluorescence quenching was observed for GT-40, a compound shown to have some effect on HBV replication (Table 1), suggesting a different binding site (away from W102) or mechanism for the inhibitory effect. Other compounds (GT-33, GT-39, GT-45, and GT-47) showed reproducible but modest fluorescence quenching that could not be fit to titration curves to generate the estimated binding constants. These compounds could therefore be binding nonspecifically or not inducing changes in tryptophan environments that give rise to substantive fluorescence quenching.^46^

### CAMs Misdirect Cp149 Assembly

**Figure 5:**
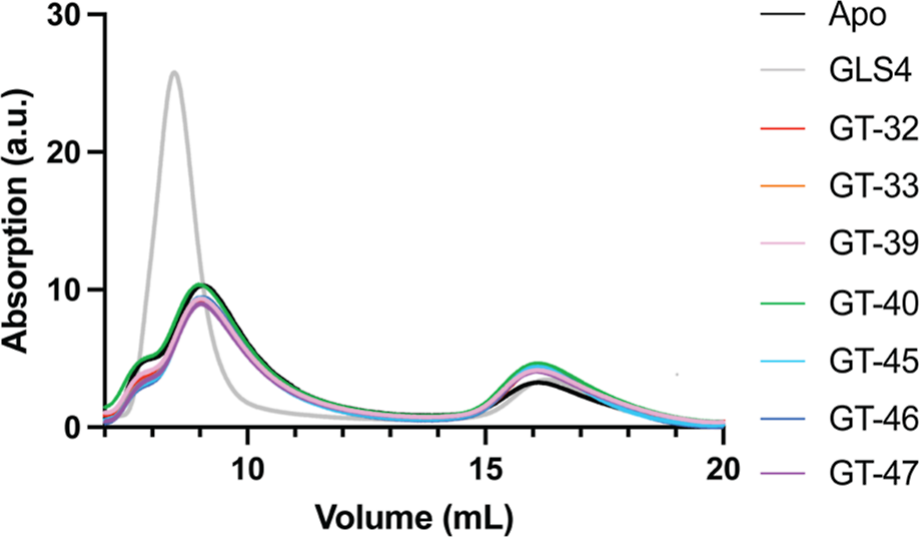
Size exclusion chromatography of Cp149 (20 μM) incubated with each CAM (40 μM) in a 500 mM NaCl solution for 1 h at 37 °C, conditions assumed from previous studies to be at equilibrium.^47^ Assembled capsids elute at ∼9 mL and dimer at ∼16 mL. “Apo” indicates no added compound.

Upon incubation with Cp149 dimers under standard assembly conditions, the CAM compounds did not induce much change in the distribution of dimers and capsids in comparison to the strong enhancement of assembly with GLS4. The novel CAMs also did not significantly affect the size of the capsids, as shown by the same elution volumes as the additive-free (Apo) protein (Figure 5).

**Figure 6:**
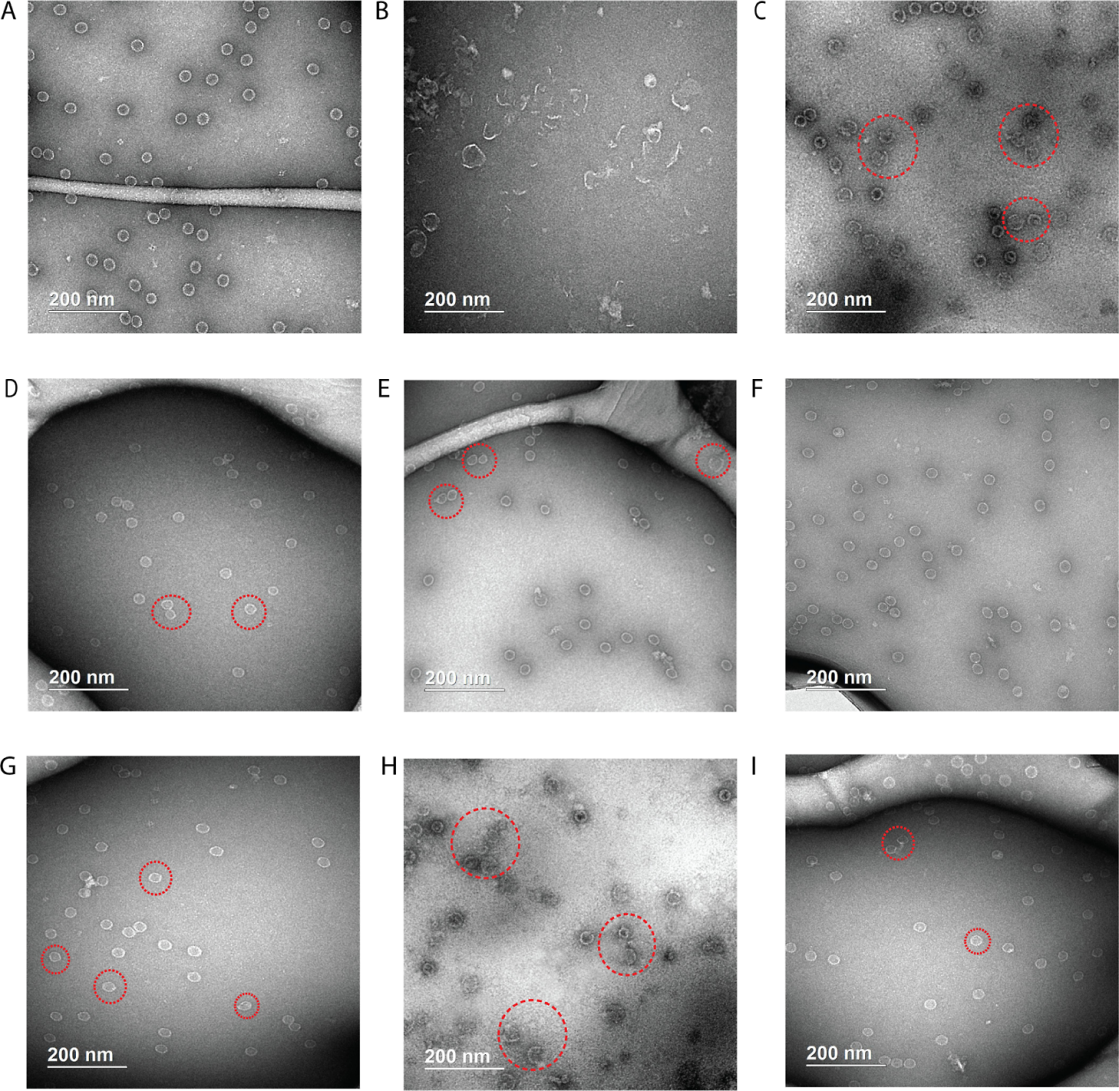
TEM images of the assembled products for the apo state (A), GLS4 (B), GT-32 (C), GT-33 (D), GT-39 (E), GT-40 (F), GT-45 (G), GT-46 (H), and GT-47 (I). The scale bar for image (B) is 0.5 μm, while the scale bars for all the other images are 200 nm. Some representative products are indicated by red circles. More images of the products for apo, GT-32, GT-39, and GT-46 are shown in Figures S9 and S10.

Transmission electron microscopy (TEM) proved to be more informative, illustrating the effects that correspond to the measured binding affinities to Cp149. Without added CAM, the assembled capsids were highly uniform and spherical in shape (Figure 6A). GLS4 (Figure 6B) showed strong misdirecting effects, giving rise to aggregates 2–5 times larger than standard capsids, a wide size distribution, and very few normal spherical structures. Although the novel CAMs did not distort capsid structures to the extent observed with GLS4, they still induced the formation of some incomplete or misshapen spherical capsids. This suggests that the CAMs act as misdirectors in the capsid assembly process, a behavior that is supported by our computational models. One exception is GT-40, which had no apparent effect (Figure 6F), so it is possible that GT-40 does not function as a CAM. Its efficacy could potentially be explained by mechanisms of action beyond capsid assembly modulation or might simply be the consequence of the inherent inconsistencies found in cellular assays. The latter situation highlights the challenges in interpreting cellular assay results, especially for compounds that do not show clear activities. The proportion of abnormal capsids was determined through the analysis of a minimum of 100 capsids, randomly selected from images corresponding to each treatment, as detailed in Table S8. This experiment was replicated, and the results were averaged to enhance the reliability. GT-32 (Figure 6C) and GT-46 (Figure 6H), which showed measurable binding constants to Cp149, induced a relatively high fraction of abnormal capsids (around 81 and 74%, respectively). GT-39 did so as well (around 41% abnormal capsids) and showed a greater degree of capsid abnormalities than the other novel CAMs (Figure 6E), indicative of direct binding despite minimal tryptophan fluorescence quenching (Figure 4). MD simulations predicted a large misdirecting effect of GT-39 when bound (Figure 3B), suggesting that this large effect may compensate for weak binding in experiments. Other possibilities, such as minimal change in the tryptophan’s environment upon GT-39 binding, also remain.

## Discussion

Biophysical methods probing the structure, dynamics, and function of target proteins and protein–ligand complexes have proven invaluable in the earliest stages of drug discovery.^48^ For example, ligand binding affinity and in vitro efficacy measurements, coupled with computational approaches such as HTS, docking, and MD have played complementary roles in preclinical stages of hit-to-lead compound design. Leveraging our earlier work^37^ on elucidating the mechanistic aspects of CAM binding on the structure and functions of HBV early assembly intermediates, we have extended the search for novel compounds using a variety of biophysical approaches along with in vitro experiments to validate these compounds and generate promising candidates for further development.

In this study, we have successfully combined molecular docking, MD simulations, biophysical, and in vitro experiments to screen large databases of compounds, and we have identified several novel lead CAMs targeting the HBV nucleocapsid. Tryptophan fluorescence measurements showed that these compounds bind directly to Cp149, and other biophysical measurements (SEC, TEM), along with MD simulations, showed that they alter the morphology of the assembled capsids, displaying moderate misdirecting effects. In vitro assays also indicated that they exhibit moderate HBV DNA inhibition and low toxicity, albeit with a high variability (Table 1). Such work is crucial in order to avoid time-consuming lead optimization on ineffective compounds. Additional tests for viral inhibition were conducted on three compounds: GT-46, which had demonstrated high efficacy relative to that of other compounds; GT-39, identified for its moderate efficacy and potential for optimization; and GT-47, which had exhibited minimal efficacy. As anticipated, the results, detailed in Table S4, continued to show variability, highlighting the limitations of cellular assays for assessing the antiviral properties of CAMs exhibiting weak inhibition.

The constraints associated with in vitro assays, particularly noted in the study of the Ebola virus by Postnikova et al. (2018),^43^ highlight the challenges posed by compounds with suboptimal efficacy. Despite these limitations, these cellular assays still offer a preliminary indication for the filtering of the compounds. However, for a more robust validation of potential therapeutic agents, it is essential that these in vitro assays be integrated with additional investigative methods. This layered approach ensures a comprehensive evaluation, enabling the advancement of the most promising compounds through the drug development pipeline. In this context, biophysical measurements, including tryptophan fluorescence, SEC, and TEM, have demonstrated consistent outcomes, as evidenced by Figure 4–6. These methods offer greater consistency because they directly explore the interactions between the compounds and the target protein, thereby facilitating a more immediate observation of the compounds’ misdirecting effects. Furthermore, these biophysical approaches mitigate the influence of numerous extraneous factors that could potentially skew results, such as those in cellular-based assays. By circumventing these variables, biophysical measurements provide a more focused assessment of compound efficacy and mechanism of action, allowing one to select the compounds most suitable for lead optimization. In addition, by incorporating computational methods like molecular docking and MD simulations, our study provided insights to the mechanisms of action of even nonoptimized compounds such as these, revealing that the selected CAMs induce changes in the conformations of early assembly intermediates, consistent with the altered capsid morphologies observed by electron microscopy.

The binding affinity and the binding pattern of the compounds are two factors affecting the efficacy of the anti-HBV compounds.^49^ The former was tested by tryptophan fluorescence titration. The two compounds with relatively higher affinity, GT-32 and GT-46, also showed good activities indicated by the higher fractions of misdirected capsids (Figure 6C,H, respectively, and Table S8). Some of the other compounds (GT-33, GT-39, GT-45, and GT-47) induced misdirected products even though no robust fluorescence quenching was observed, which can still be consistent with direct binding to the Cp149 pocket. Docked structures of GT-32, GT-39, and GT-46 occupy a space in the pocket similar to GLS4 and, like other HAPs, form a hydrogen bond with W102 (Figure S12).^37^

The case of GT-39 provided an interesting example of the value of computational simulations in the discovery process. MD simulations focused on how the binding of CAMs affects the orientations of early assembly intermediates. These simulations suggested that the GT-39-bound tetramer should adopt a relatively large deviation from the structure of the apo hexamer, similar to the conformations induced by the potent misdirector GLS4. And, indeed, GT-39 induced a higher fraction and greater degree of capsid abnormality compared to those of other novel CAMs (Figures 6E and S10). However, its relatively low binding affinity made its overall misdirecting inferior to that of GLS4. In the case of GLS4, which has a *K*_D_ of 41 μM, almost half of the dimers were bound by GLS4 under the experimental concentration of 20 μM Cp149 and 40 μM compound. Consequently, all of the assembled capsids contained a large fraction of GLS4-bound, and thus misdirected, tetramers. As seen in the TEM image in Figure 6B, almost all of the assembled products were abnormal compared to the apo group, some of which completely lost the spherical morphology of normal capsids. In comparison, with much weaker binding affinity than GLS4, GT-39 could only affect a smaller fraction of protein, giving rise to a smaller fraction (around 41% based on TEM images; Table S8) of abnormal capsids.^50^ Despite GT-39’s relatively weak binding affinity, it consistently demonstrated moderate antiviral effects in the majority of trials, as evidenced by the results of cellular assays presented in Tables 1, S3 and S4. This suggests that GT-39 has potential as an antiviral agent, especially if its binding affinity can be enhanced through additional optimization.

Furthermore, it is worthwhile to compare the performance of our current most promising compounds to those of established CAMs in the literature. Reported dissociation constants for GLS4 (41 μM), GLP-26 (0.7 μM),^27^ and several HAP compounds (≪3 to 20 μM)^6^ indicate overall weaker binding by our current compounds. Interestingly, despite the observed misdirection in capsid assembly (Figure 6), SEC analysis revealed a minimal impact on the overall size distribution of Cp protein assemblies (Figure 5). This implies that the compounds might only induce localized changes in the assembly process while preserving the general spherical configuration of the capsids, even in the misdirected ones. Nonetheless, the aberrant capsids and modest HBV DNA inhibition coupled with low cytotoxicity establish the current series, particularly GT-32 and GT-46, as attractive candidates for future lead optimization.

Finally, it is worth emphasizing that at this early stage of drug discovery endeavors, the principal objective lies in the identification of the compounds that demonstrate specific interactions with the target protein, altering it in some way. An illustrative example is the study by Ghahremanpour et al.,^51^ which identified the lead compounds for the main protease of severe acute respiratory syndrome coronavirus 2 through a combination of virtual screening and experimental testing. Despite these compounds being suboptimal and requiring relatively high concentrations, they reveal essential molecular-level insights, setting a foundation for further research. Building on this in a follow-up study, Zhang et al.^52^ optimized these leads, discovering compounds with significantly enhanced antiviral activities, as confirmed by cell-based assays. In the context of our study, the crucial interactions and their effects on capsid assembly were observed through biophysical assays, validating the predictions from computational modeling. This approach led to the identification of promising compounds notwithstanding the variability of cellular assays. As our research progresses into lead optimization utilizing the insights gained from these studies, the determination of accurate EC_50_ values to assess antiviral efficacy will become paramount. This transition underscores the iterative nature of drug development, where initial discoveries provide the basis for the ongoing refinement and optimization of compounds. Such a process progressively guides us toward identifying viable candidates for clinical development.

## Conclusions

By combining computational methods and experimental testing, we have discovered several novel compounds targeting HBV, with moderate antiviral activities and good safety profiles. The experimentally observed capsid misdirecting effects aligned, at least in part, with computational modeling and predictions that focused on conformations of early assembly intermediates. We propose that the potency of the CAMs is determined by both their ability to alter interdimer orientations and their affinity for the capsid protein. Our work illustrates a rational, computational, and experimental approach to identifying and validating promising compounds in a preclinical setting with lead optimization to follow. Future work will focus on optimizing the selected leads to improve their affinities and performance, potentially opening up new avenues for HBV inhibition.

## Methods

### Molecular Dynamics

AMBER16^53^ and the CHARMM36 protein force field^54^ were used for all simulations. The CGenFF force-field^55^ parameters for the novel compounds were obtained from the CGenFF web server.^56,57^ The starting point in each simulations was the Tetra2 protein structure, selected from the MD trajectories generated in our previous work,^37^ with corresponding docked compounds. In each case, a Cp149 dimer or dimers with bound compound was solvated and ionized with 0.15 M NaCl using solvate and ionize plugins in VMD (Figure S13). The size of each system was approximately 135,000 atoms. In our MD protocol, we used rigid bonds for all covalent hydrogen bonds, allowing us to integrate the equations of motion with a 2 fs time step. van der Waals interactions were cutoff at 12 Å, with a smoothing function applied from 10 to 12 Å to ensure a smooth decay to zero. The particle-mesh Ewald method^58^ was used for long-range electrostatic interactions. The temperature and the pressure were kept constant at biologically relevant values of 310 K and 1 bar, respectively. We used a Langevin thermostat for temperature control and a Berendsen barostat with τ = 1.0 ps for pressure control. The energies of all systems were minimized in two steps before equilibration. In the first energy minimization step, only water and ion positions were optimized, while the protein and CAM were restrained. In the second step, the positions of all of the atoms were optimized. After minimization, a two-step equilibration was performed for all of the systems. First, water and ions were equilibrated for 0.5 ns while restraining the protein and the CAM. In the second 1 ns-long step, only the protein backbone was restrained. A harmonic force constant of 2 kcal/mol·Å^2^ was used for the restraint in all cases. After equilibration, two 150 ns long simulations were performed for each system.

### Analysis of Simulations

The first 10 ns of each production run was discarded prior to analysis, after which the trajectory frames were analyzed at a frequency of 0.5 ns. The definitions for base and spike angles were taken from previous work. For each system, the data from 2 × 150 ns simulations are combined and projected on a 2D scatter plot. The SDEs were drawn for each system to enable an easier comparison of the sampled structures. In a 2D plot, the SDEs are centered at the average values of the two variables, while the relative height and width are determined by the standard deviations of these variables.^44^ The rotation of the SDE is calculated from variable correlation, and the total ellipse size is scaled to encompass a specific percentage of the provided distribution,^44^ which corresponds to the confidence level of the ellipse. We chose to plot the ellipses corresponding to a 90% confidence level.^44^

### Docking

All docking runs were done with Glide from Schrödinger with the default settings.^59,60^ We used Glide high-throughput virtual screening (HTVS) for the initial screening on the ZINC0.9 library,^42,59^ which contained ∼100,000 compounds. The top 20,000 highest scoring compounds were selected for redocking with Glide single precision (SP), and the top 5000 of those compounds were redocked with Glide extra precision (XP).^61^ For the DivV library, we first used Glide SP to dock all ∼5000 ligands and selected the top 1000 high-scoring ligands for redocking with Glide XP. During optimization of our hit compound, we only needed to dock around 2000 compounds and, therefore, only used Glide XP.^59,61^ When selecting the top scoring molecules, compounds with expected low solubility or high reactivity and toxicity due to the presence of certain chemical groups were discarded. Molecules that contained PAINS groups^62^ or were not readily available to order were excluded as well. Finally, we aimed for structural variance and selected compounds from different databases for testing.

### Inhibition Assays

HepAD38 cells were seeded at 50,000 cells/well in collagen-coated 96-well plates. Test compounds were added to HepAD38 cells in a dose–response manner up to a final concentration of 10 μM. The experiment lasted 7 days. On day 7, total DNA was purified from the supernatant using a commercially available kit (DNeasy 96 Blood & Tissue kit, Qiagen). The HBV DNA was amplified in a polymerase chain reaction assay using LightCycler 480 II (Roche), as previously described.^63^ All samples were tested in duplicate. The concentration of the compound that inhibited HBVDNA replication by 50% (EC_50_) was determined by linear regression.

### Cytotoxicity Assays

Human peripheral blood mononuclear (PBM), T lymphoblast CEM-CCRF (herein referred to as CEM cells), African green monkey kidney (Vero), or human liver carcinoma (HepG2) cells were tested via MTT assay using the CellTiter 96 Non-Radioactive Cell Proliferation (Promega) kit as previously described.^63^ Cytotoxicity was expressed as the concentration of test compounds that inhibited cell proliferation by 50% (CC_50_) and calculated using the Chou and Talalay method.^64^

### Preparation of Cp149 Protein

Recombinant production of Cp149 proteins was performed in *E. coli* BL21(DE3) cells (New England Biolabs). A single transformant colony containing the pET11a:Cp149 plasmid was first inoculated into 500 mL of 2YT growth medium supplemented with 0.1 mg/mL carbenicillin in a 2 L Erlenmeyer flask. The cell culture was then grown to saturation for 24 h at 26 °C in a shaking incubator set to 250 rpm before harvesting the cells via centrifugation in a JA-16.250 rotor (Beckman Coulter) at 8000 rpms for 15 min at 4 °C. The supernatant was discarded, and the resulting cell pellets were stored at −80 °C until purification.

Purification of recombinant Cp149 particles was achieved by first resuspending the cell pellets from the entire 500 mL expression culture in 100 mL of 50 mM potassium phosphate (pH 7.5) and 0.5 M NaCl on ice. The resuspended cells were lysed by sonicating the cells in 5 s pulses (50 W per pulse) with 5 s of rest between the pulses for a total of 10 min. The cell lysate was subsequently clarified by centrifugation at 14,000 rpm in a JA-17 rotor (Beckman Coulter) for 30 min at 4 °C. The VLPs were then precipitated from the clarified lysate by adding solid (NH_4_)_2_SO_4_ to a final concentration of 30% (w/v) and incubating the sample at 4 °C for 1 h. The resulting solid precipitates were collected by another centrifugation at 14,000 rpm in a JA-17 rotor for 30 min at 4 °C. The supernatant was discarded, and then, the solid precipitate pellets were resuspended overnight at 4 °C in 30 mL of 50 mM potassium phosphate (pH 7.5) with gentle agitation. Residual insoluble materials were removed by centrifugation at 14,000 rpm in a JA-17 rotor for 15 min at 4 °C. The supernatant was collected, and the resuspended VLPs were concentrated from the supernatant by ultracentrifugation at 68,000 rpm in a Type 70-Ti rotor (Beckman Coulter) for 2 h at 4 °C. The supernatant was decanted, and the resulting VLP-containing pellet was resuspended overnight at 4 °C in 12 mL of fresh 50 mM potassium phosphate buffer (pH 7.5) with gentle agitation. The resuspended VLP sample was then further purified by 10–40% (w/v) sucrose gradient sedimentation at 28,000 rpm in a SW32 rotor (Beckman Coulter) for 4 h at 4 °C. The VLPs were extracted from the sucrose gradients via aspiration and were concentrated in a final ultracentrifugation step at 68,000 rpm for 2 h in a Type 70-Ti rotor. The resulting protein pellet was resuspended overnight in 5 mL of 50 mM sodium bicarbonate (pH 9.6) and 2 mM DTT buffer at 4 °C with gentle agitation.

Purified VLPs were disassembled into Cp149 dimers by adding 2.09 g of solid urea per 10 mL of aqueous sample and incubating the sample on ice for 90 min in accordance with previously published protocols.^65^ The sample was then transferred to a 7 kDa molecular weight cutoff cellulose dialysis bag and was dialyzed overnight at 4 °C against a solution of 50 mM *N*-(2-hydroxyethyl)piperazine-*N′*-ethanesulfonic acid (HEPES) (pH 7.5) and 5 mM dithiothreitol (DTT). The final concentration of the purified Cp149 protein was determined using a Coomassie Plus Bradford Assay Kit (Pierce) with bovine serum albumin as the protein standard. The purity of the final product was verified via denaturing electrophoresis on a Bioanalyzer 2100 system (Agilent Technologies) by using a Protein 80 microfluidic electrophoresis assay chip.

### Native Agarose Gel Electrophoresis

Native agarose gel electrophoresis of the disassembled vs reassembled VLPs was performed by loading 10 μg of protein sample per lane in a 1.5% agarose gel prepared in 0.5× tris-acetate-EDTA (TAE) buffer. Electrophoresis was performed at a constant potential of 150 V for 30 min in a 0.5× TAE buffer. Proteins were subsequently visualized by immersing the gel in staining solution (0.05% w/v Coomassie brilliant blue powder dissolved in an aqueous solution of 25% v/v isopropanol and 10% v/v acetic acid) for 10–15 min, followed by immediate destaining of the gel for a minimum of 4 h in 10% v/v acetic acid.

### Size Exclusion Chromatography

The Cp149 protein solution and the compound solution were initially mixed in concentrations of 40 μM Cp149 and 80 μM compound and incubated for 1 h at 37 °C to allow the compound to bind to the protein. The NaCl solution was then added to the incubated mixture to final concentrations of 20 μM Cp149, 40 μM compound, and 500 mM NaCl to initiate the assembly of Cp149. This mixture was incubated at 37 °C for 1 h. Finally, 100 mL of the sample was analyzed using a hand-poured Superose 6 column, at the flow rate of 0.4 mL/min, and the isocratic mobile phase was 50 mM HEPES (pH 7.5). All the samples were run in triplicate, and a representative curve from the three replicates is shown in Figure 5.

### Tryptophan Fluorescence Assays

Cp149 protein was diluted with 50 mM HEPES buffer to the final concentration of 20 μM. Each of the compounds was titrated into 100 μL of the protein solution to final concentrations of 0, 5, 10, 20, 50, and 100 μM in a clear bottom 96-well plate. Blank control samples were prepared by titrating the compounds into 50 mM HEPES buffer without Cp149. Tryptophan fluorescence levels were measured by exciting the samples at 285 nm and measuring the emission at 340 nm with the Varioskan Flash (Thermo Fisher) plate reader. The net fluorescence levels were obtained by subtracting the blank controls from the corresponding Cp149 samples to rule out the intrinsic fluorescence from the compounds. Fluorescence quenching was calculated by taking the difference of net fluorescence levels of each concentration and of the 0 μM sample. All the samples were run in triplicate, and the results were averaged. The dissociation constants (*K*_D_) were fitted by the one-site-specific binding model of GraphPad.^45^

### Transmission Electron Microscopy

The assembled samples of Cp149 were prepared as described in the Size Exclusion Chromatography section. Eight μL of each sample was added onto the 300 mesh Lacey Formvar carbon–copper grids (Ted Pella, Inc.) and allowed to sit for 1.5 min. The grid was then rinsed in 500 mL of deionized water for 10 s twice to rinse off the extra buffer on the grid. After drying, the sample was stained with 8 μL of 2% uranyl acetate for 1 min. After removal and drying of the uranyl acetate solution, the sample was imaged with a Hitachi HT7700 electron microscope at an accelerating voltage of 120 kV, at the IMat Materials Characterization Facility of Georgia Institute of Technology. The images underwent processing using ImageJ software. Initially, to minimize noise, a Gaussian blur filter was applied. These images were then converted into an 8 bit binary format for edge detection. ImageJ identified particles ranging in size from 800 to 2000 pixels. An example of a processed image can be seen in Figure S14. The circularity of each particle was calculated using the formula circularity = 4π × area × perimeter^–2^. Capsids that either could not be detected by ImageJ or exhibited a circularity below 0.85 were categorized as abnormal. A total of four images, each containing a minimum of 100 capsids, were analyzed to determine the proportion of the abnormal capsids. The entire experiment was duplicated, and the outcomes from both sets were then averaged.

## Supporting Information Available

Additional information about MD simulations, 2D plot of PCA analysis, structures of compounds, BioAnalyzer data, DLS data, NAGE data, more TEM images, illustration of the docking structures, summarized results of HBV DNA inhibition and toxicity testing, range of sampled base and spike angles from MD simulations, and FOAs for SDEs of spike and base angles from MD simulations (PDF)

## Abbreviations

HBV: hepatitis B virus
Cp: capsid protein
CAM: capsid assembly modulator
MD: molecular dynamics
DNA: deoxyribonucleic acid
cccDNA: covalently closed circular DNA
pgRNA: pregenomic RNA
HAP: heteroaryldihydropyrimidine
PPA: phenylpropenamide
SBA: sulfamoyl benzamide
cryo-EM: cryogenic electron microscopy
NMR: nuclear magnetic resonance
HTS: high-throughput screening
PCA: principal component analysis
PBM: peripheral blood mononuclear
CEM: T lymphoblast CEM-CCRF
Vero: African green monkey kidney
HepG2: human liver carcinoma
CC50: 50% cellular cytotoxicity concentration
SDE: standard deviational ellipse
FOA: fractional overlap area
SEC: size exclusion chromatography
VLP: virus-like particle
TEM: transmission electron microscopy
PAINS: pan-assay interference compounds.
