# Supplementary material for: Biophysics-Guided Lead Discovery of HBV Capsid Assembly Modifiers
Source: ACS Infect Dis. 2024 Apr 2;10(4):1162–73. doi: 10.1021/acsinfecdis.3c00479 (PMC11019538; doi:10.1021/acsinfecdis.3c00479)
Supplement: Supplementary file 1 — id3c00479_si_001.pdf [file id3c00479_si_001.pdf]

Supporting Information

Biophysics-Guided Lead Discovery of HBV Capsid Assembly Modifiers

Zixing Fan,[†] Anna Pavlova,[‡] Matthew C. Jenkins,[¶] Leda Bassit,[§] Mohammad Salman,[§] Diane L. Lynch,[‡] Dharmeshkumar Patel,[§] Maksym Korablyov,[‖] M. G. Finn,[¶] Raymond F. Schinazi,[§] and James C. Gumbart[*,‡,¶]

†*Interdisciplinary Bioengineering Graduate Program, Georgia Institute of Technology, Atlanta, GA, 30332 USA*

‡*School of Physics, Georgia Institute of Technology, Atlanta, GA, 30332 USA*

¶*School of Chemistry & Biochemistry, Georgia Institute of Technology, Atlanta, GA, 30332 USA*

§*Center for ViroScience and Cure, Laboratory of Biochemical Pharmacology, Department of Pediatrics, Emory University School of Medicine and Children's Healthcare of Atlanta, Atlanta, GA, 30322 USA*

‖*MIT Media Lab, Massachusetts Institute of Technology, Boston, MA, 02139 USA*

* E-mail: gumbart@physics.gatech.edu

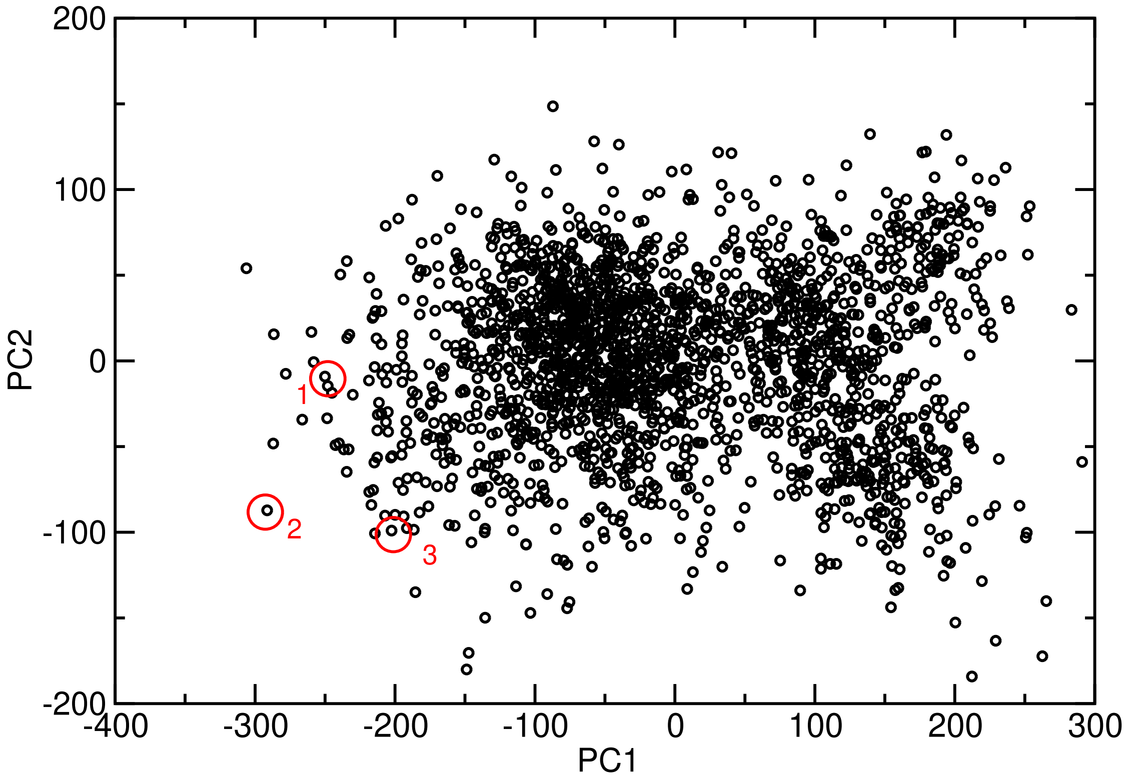

Figure S1: 2D plot of the two largest principal components in our PCA analysis of apo tetramer. The structures that were selected for docking are marked in red.

1

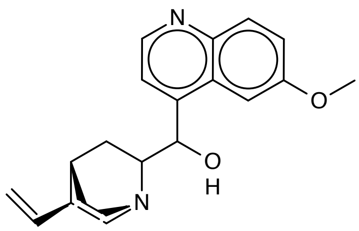

2

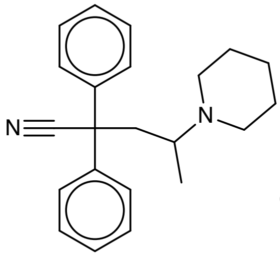

3

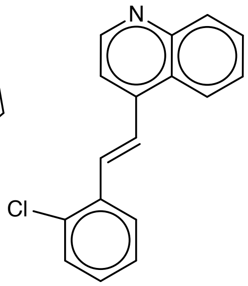

4

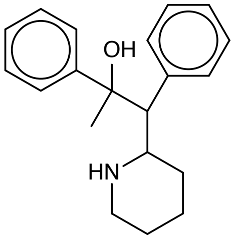

5

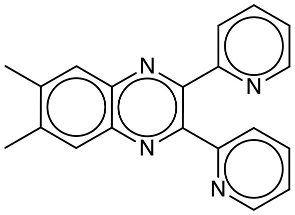

6

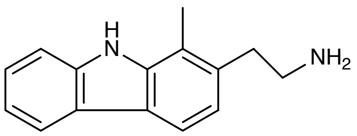

7

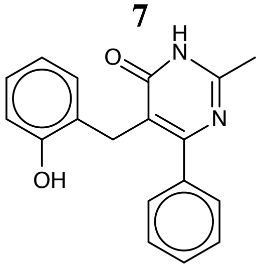

8

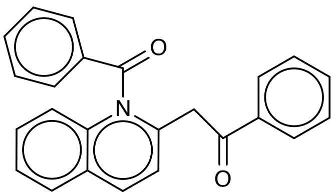

9

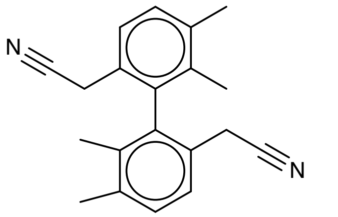

10

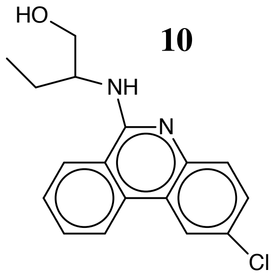

11

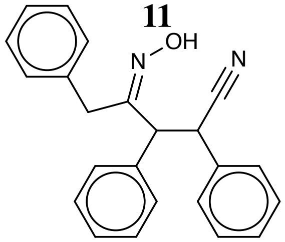

Figure S2: Structures of compounds 1-11.

12

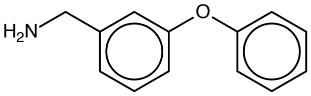

13

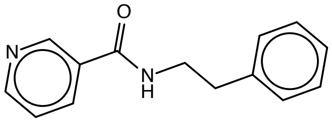

14

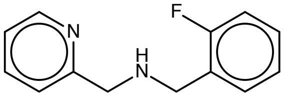

15

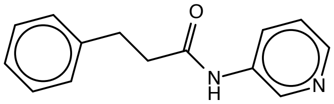

16

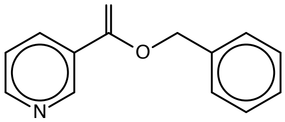

17

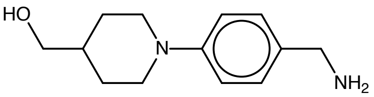

18

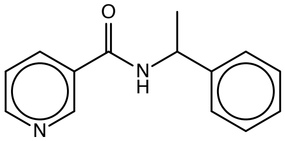

19

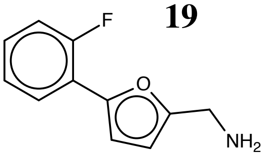

20

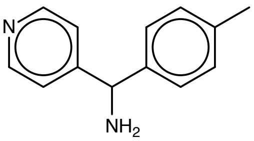

21

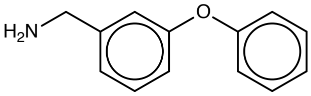

22

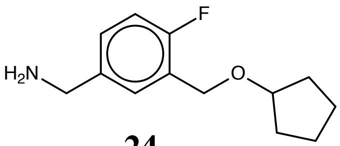

23

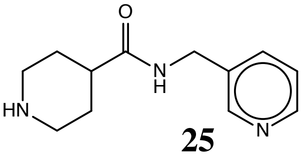

24

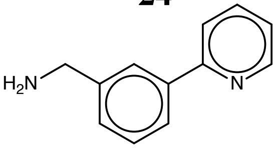

25

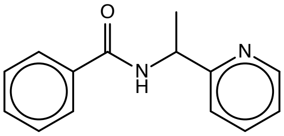

26

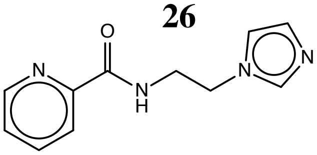

27

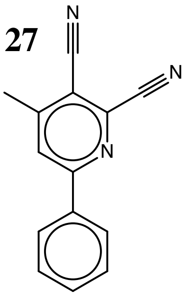

28

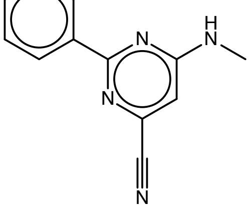

29

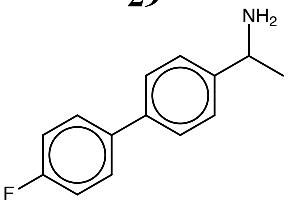

Figure S3: Structures of compounds 12-29.

30

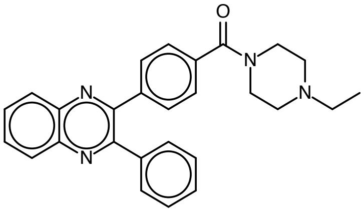

31

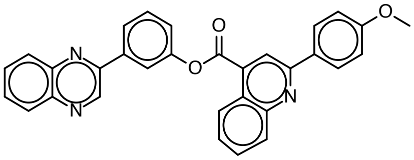

32

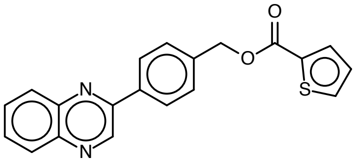

33

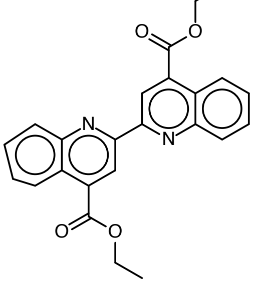

34

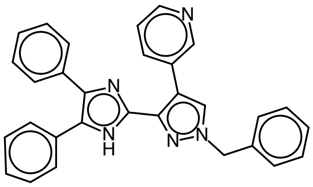

35

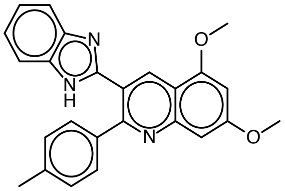

36

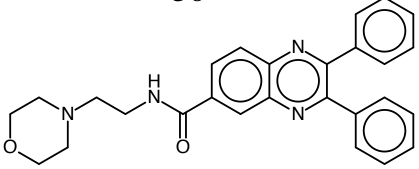

37

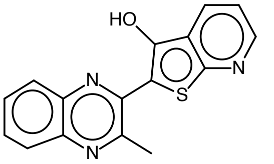

38

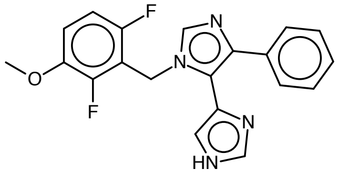

39

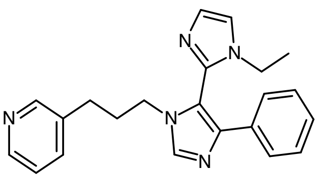

40

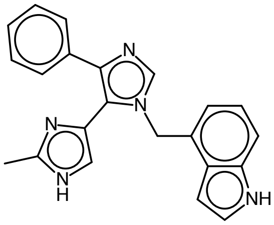

41

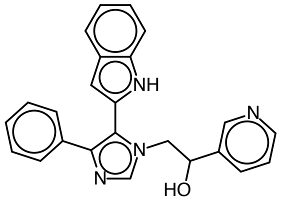

Figure S4: Structures of compounds 30-41.

42

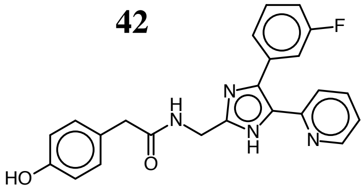

43

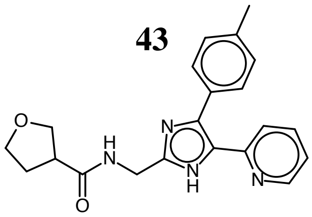

44

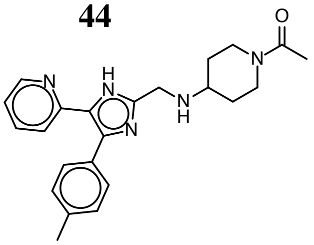

45

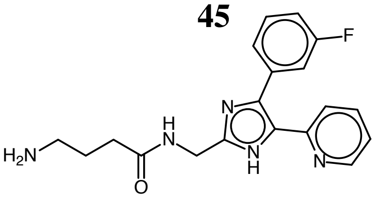

46

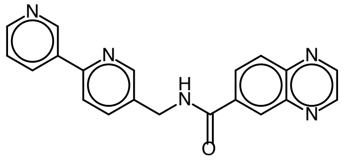

47

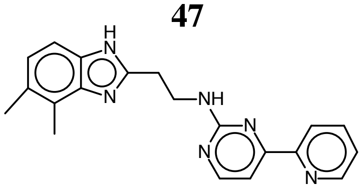

48

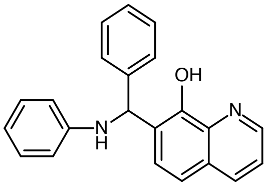

49

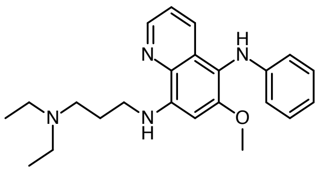

Figure S5: Structures of compounds 42-49.

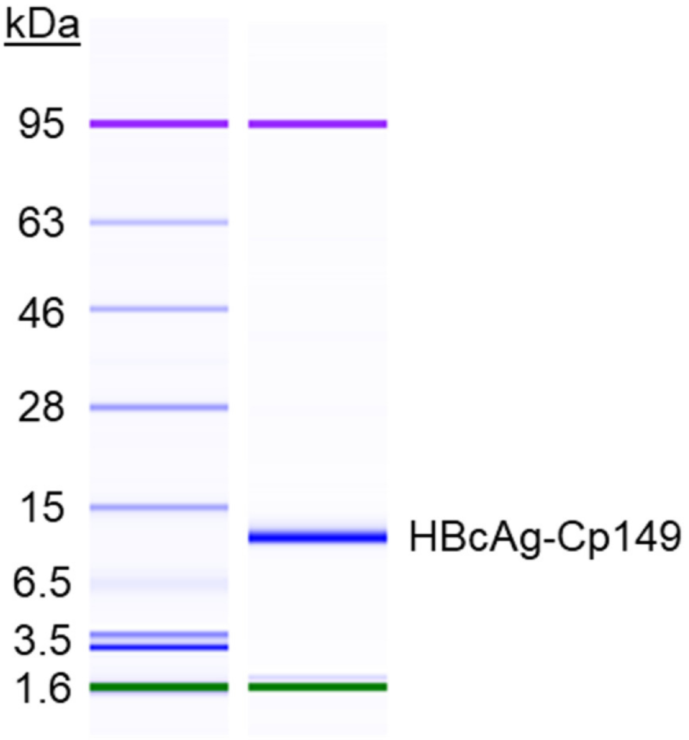

Figure S6: BioAnalyzer data of the purified Cp149 protein indicating that the sample is clean. The Cp149 protein band migrates lower than its anticipated monomer MW (16.8 kDa) in the Bioanalyzer plot, but this is likely due to it having a theoretical pI of 4.9 at physiological pH.

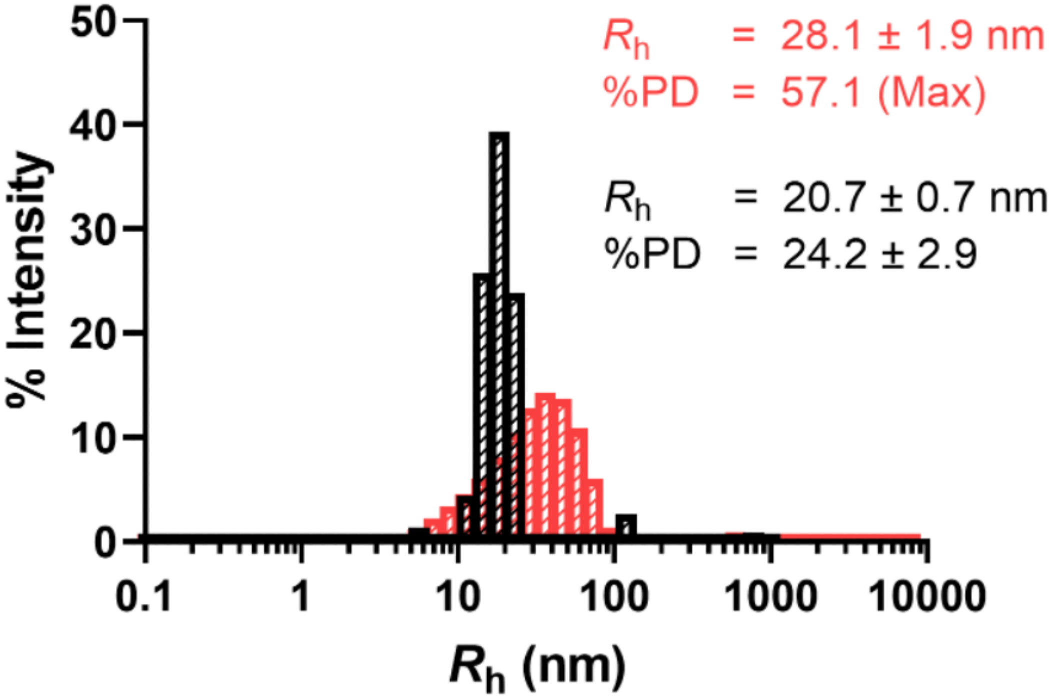

Figure S7: Dynamic light scattering (DLS) data for the disassembled (black) and assembled (red) Cp149 dimers.

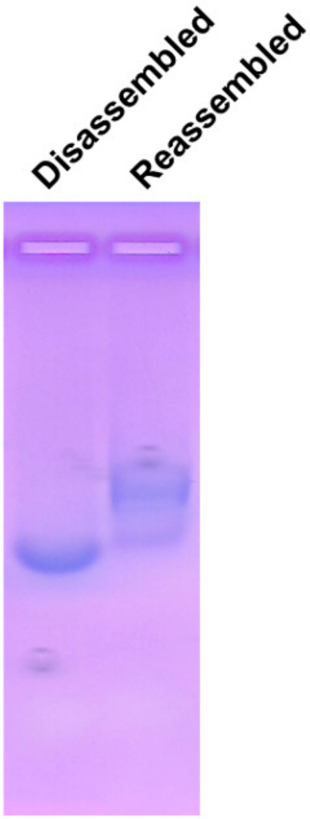

Figure S8: Native agarose gel electrophoresis (NAGE) data for the disassembled Cp149 dimers.

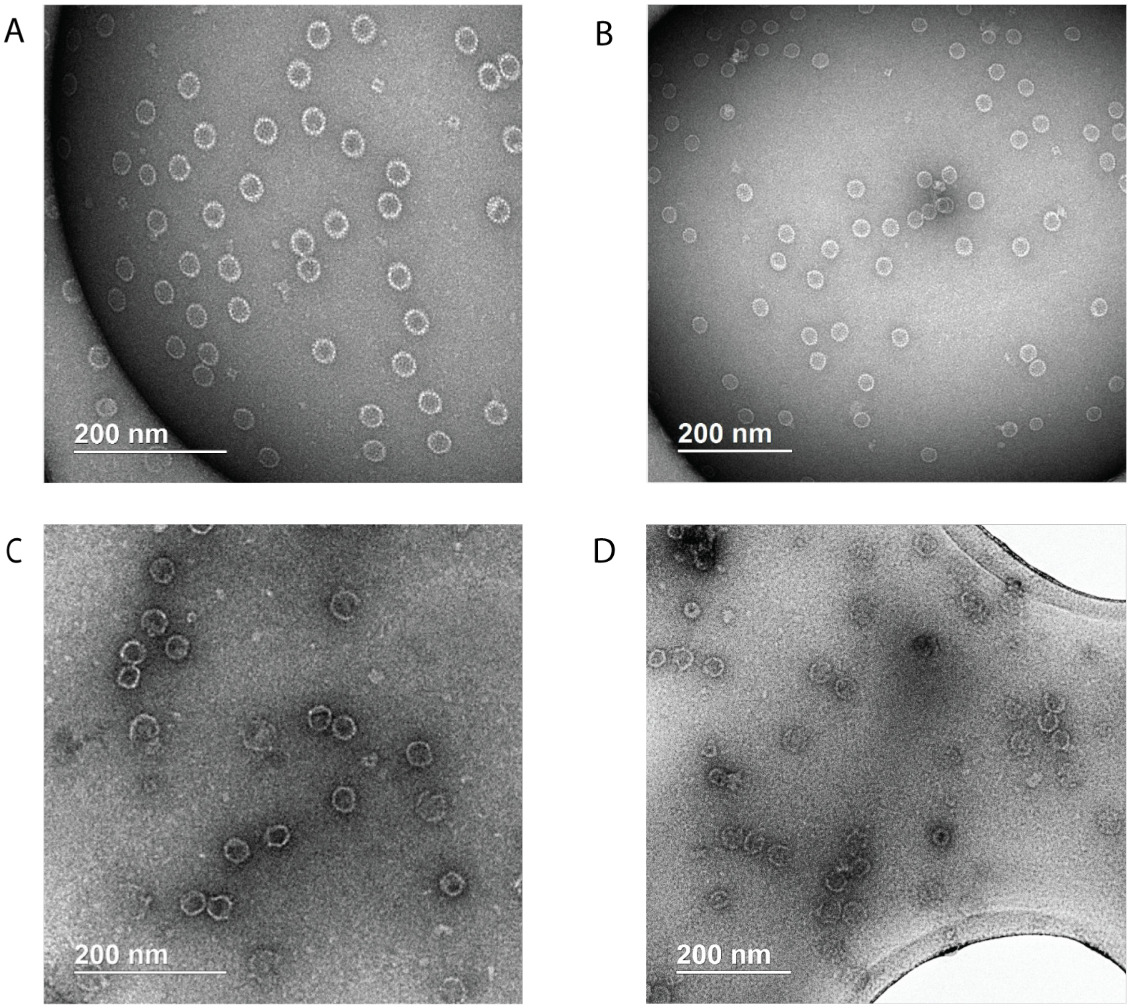

Figure S9: TEM images of assembled products without compound (A,B), and with GT-32 (C,D), with one image from each replica.

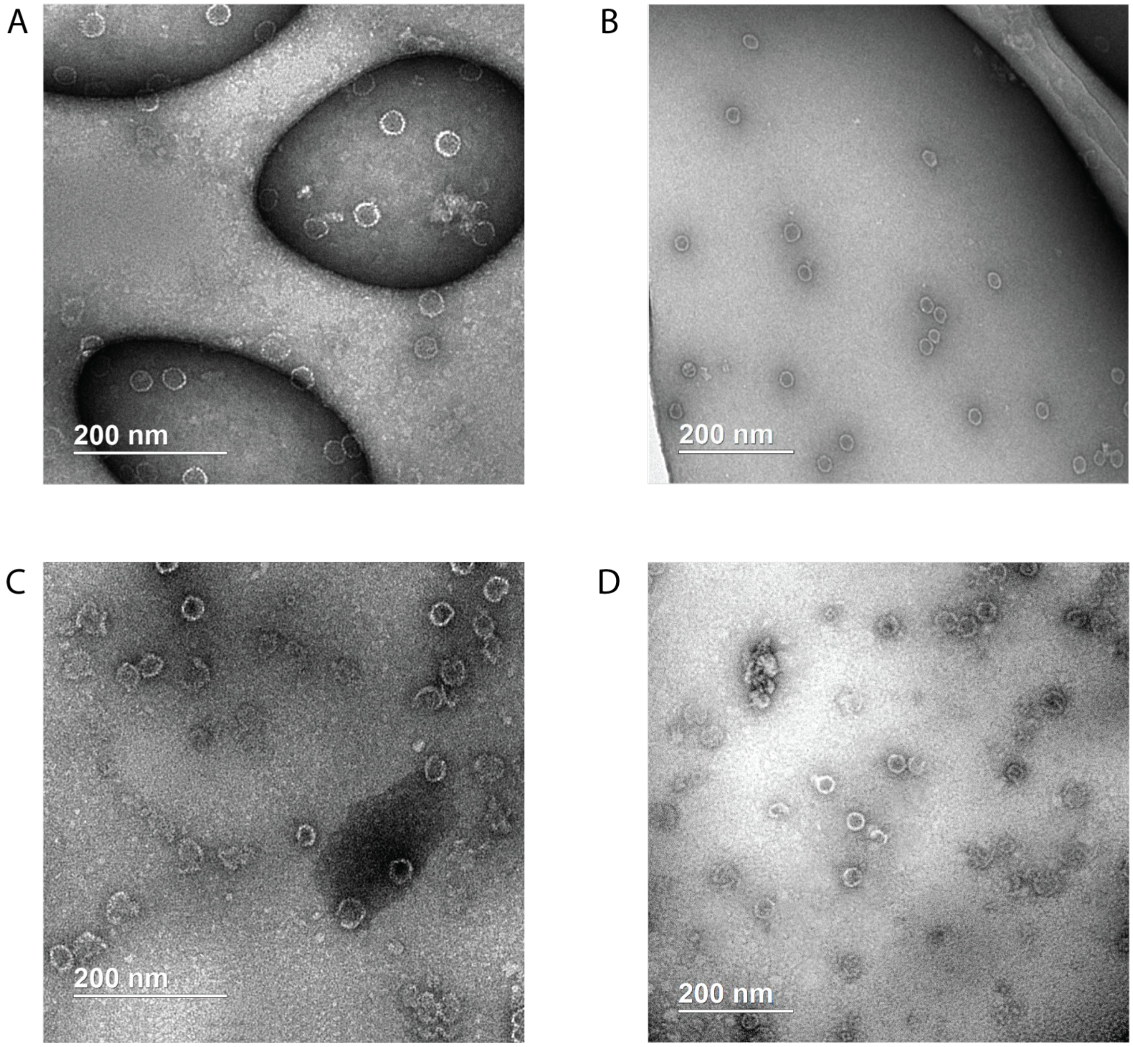

Figure S10: TEM images of assembled products with GT-39 (A, B), and GT-46 (C, D), with one image from each replica.

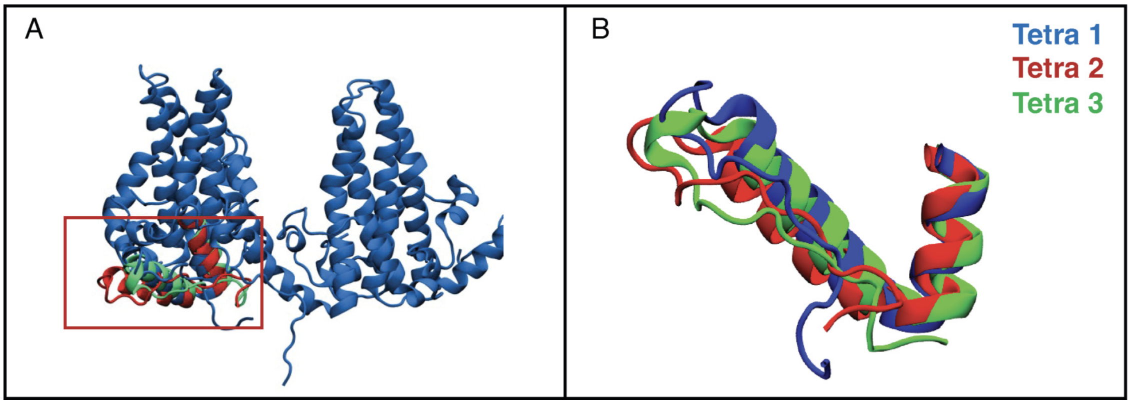

Figure S11: Illustration of the differences between the three structures used for docking. A) Tetra 1 structure (blue) with the red rectangle pointing to the most distinct region in the binding pocket between the 3 structures. Differences in Tetra 2 and Tetra 3 structures after backbone alignment are shown in red and green, respectively. B) Structural comparison of the distinct region.

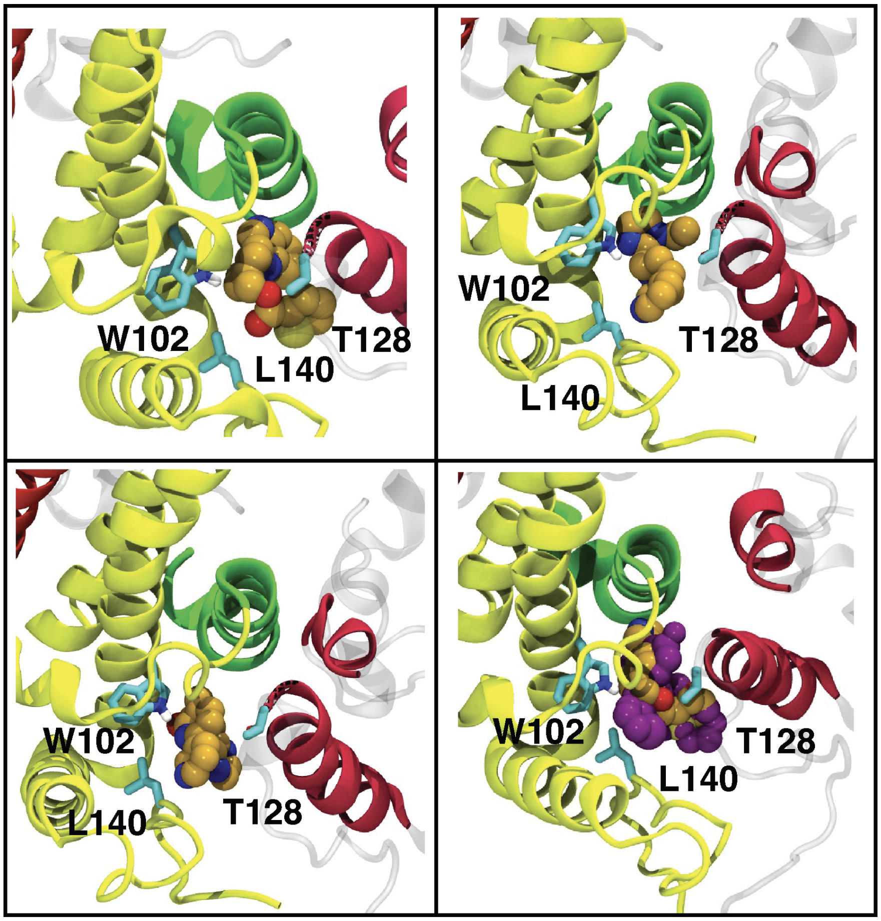

Figure S12: Illustration of docked structures for the most promising compounds in comparison to GLS4. Top Left: GT-32, top right: GT-39, bottom right: GT-46, bottom left comparison of GLS4 (purple) and GT-32 binding. The compounds are shown in the VdW representation. Carbon, oxygen and nitrogen of the compounds are shown as orange, red and blue, respectively. Three residues known to form interactions with CAMs are also displayed in cyan: W102, T128 and L140. Most hydrogens are omitted for clarity.

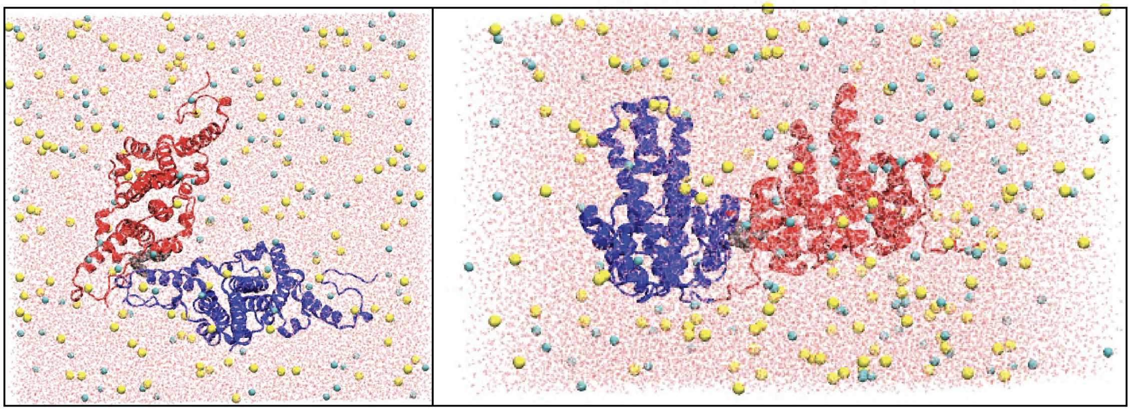

Figure S13: Example of a simulated system, the periodic box of the starting state of dimer of dimers with bound GT-32 it shown in top view (left), and side view (right). The AB dimer and the CD dimer are shown in blue and red, respectively, using cartoon representation. Water molecules are shown as red dots. GT-32 is colored gray and is shown in vdW representation. Finally ions are shown as spheres, with yellow and cyan corresponding to sodium and chloride ions, respectively.

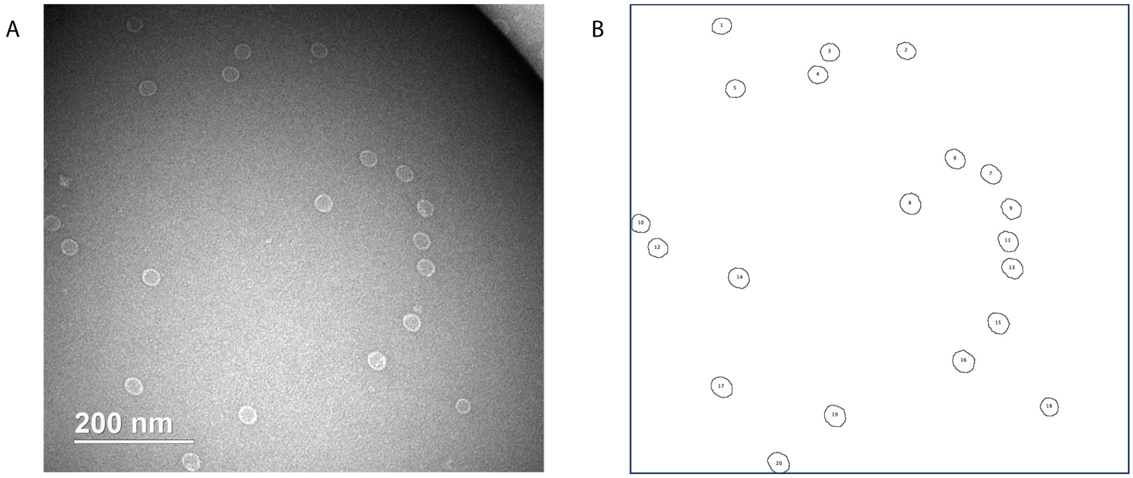

Figure S14: Illustration of an image processed using ImageJ. A) The original, unprocessed image. B) Image highlighting particles identified by ImageJ, ranging in size from 800 to 2000 pixels.

Table S1: Summarized results of HBV DNA inhibition and toxicity testing for the first round of selected compounds. N/A stands for not applied.

Comp.	HBV DNA inhibition	MTT cytotoxicity, CC_{50} (μM)			
	(%) at 10 μM	PBM	CEM	Vero	HepG2
GT-1	<1	82.9	26.9	>100	12
GT-2	<1	85.3	16.1	>100	26.4
GT-3	40.4	34.0	15.4	27.0	51.2
GT-4	<1	>100	>100	>100	>100
GT-5	24.7	>100	>100	>100	>100
GT-6	27.2	47.7	18.7	42.6	14.9
GT-7	<1	>100	>100	>100	>100
GT-8	<1	>100	>100	>100	>100
GT-9	34.6	>100	>100	>100	55.3
GT-10	<1	18.2	15.2	14.4	12.9
GT-11	<1	63.9	13.3	18.9	31.6
GT-12	<1	>100	17.0	>100	>100
GT-13	<1	>100	>100	>100	>100
GT-14	<1	>100	>100	>100	>100
GT-15	<1	>100	>100	>100	>100
GT-16	1.7	>100	>100	>100	>100
GT-17	<1	>100	>100	>100	>100
GT-18	<1	>100	>100	>100	>100
GT-19	<1	>100	>100	>100	>100
GT-20	<1	>100	>100	>100	>100
GT-21	<1	>100	18.2	49.6	>100
GT-22	<1	>100	36.3	88.6	>100
GT-23	<1	>100	>100	>100	>100
GT-24	<1	>100	11.3	>100	>100
GT-25	<1	>100	>100	>100	>100
GT-26	<1	>100	>100	>100	>100
GT-27	<1	14.7	4.0	27.3	41.7
GT-28	<1	24.0	63.8	>100	>100
GT-29	<1	25.0	35.7	21.0	12.3
3TC	97	42.4	22.5	>100	>100
Cycloheximide	n/a	0.9	0.2	0.2	0.3

Table S2: Summarized results of HBV DNA inhibition and toxicity testing for the second round of selected compounds.

Comp.	Anti-HBV activity in HepAD38 cells		MTT cytotoxicity			
	% HBV DNA Inh. at 10 μM (EC_{50} in μM)		CC_{50} in μM (% HBV DNA Inh. at 100 μM)			
	Trial 1	Trial 2	PBM	CEM	Vero	HepG2
GT-30	<1 (> 10)	<1 (> 10)	60	32	81	34
GT-31	<1 (> 10)	<1 (> 10)	86	>100 (36%)	97	42
GT-32	48 (≥ 10)	<1 (>10)	85	52	44	59
GT-33	60 (< 10)	37 (> 10)	>100 (5.6%)	>100 (32%)	76	86
GT-34	<1 (> 10)	<1 (> 10)	59	11	14	16
GT-35	36 (> 10)	36 (> 10)	>100 (14%)	>100 (49%)	42	63
GT-36	11 (> 10)	11 (> 10)	10	18	31	29
GT-37	40 (> 10)	40 (> 10)	>100 (48%)	>100 (<1%)	19	24
GT-38	45 (> 10)	45 (> 10)	>100 (24%)	62	48	24
GT-39	57 (< 10)	50 (10)	>100 (22%)	16	>100 (47%)	>100 (32%)
GT-40	55 (< 10)	50 (10)	>100 (19%)	77	>100 (39%)	>100 (30%)
GT-41	<1 (> 10)	<1 (> 10)	27	12	18	10
GT-42	<1 (> 10)	<1 (> 10)	3.5	1.4	13	5.4
GT-43	4.7 (> 10)	4.7 (> 10)	42	30	19	29
GT-44	37 (> 10)	37 (> 10)	64	63	>100 (54%)	70
GT-45	50 (≥10)	49 (≥ 10)	>100 (41%)	>100 (36%)	66	>100 (41%)
GT-46	49 (≥ 10)	49 (≥ 10)	>100 (20%)	38	53	82
GT-47	49 (≥ 10)	49 (≥ 10)	>100 (16%)	>100 (48%)	>100 (31%)	18
GT-48	<1 (> 10)	<1 (> 10)	20	3.4	4.5	34
GT-49	45 (> 10)	45 (> 10)	27	9.4	16	17
3TC	82		42	22	>100	>100

Table S3: Summarized results of HBV DNA inhibition testing for the seven selected compounds across all four trials.

Comp.	% HBV DNA Inh. at 10 μM (EC$_{50}$ in μM)			
	Trial 1	Trial 2	Trial 3	Trial 4
GT-32	48 (≥ 10)	<1 (>10)	< 1 (> 10)	< 1 (> 100)
GT-33	60 (< 10)	37 (> 10)	< 1 (> 10)	< 1 (> 100)
GT-39	57 (< 10)	50 (10)	< 1 (> 10)	36 (> 100)
GT-40	55 (< 10)	50 (10)	< 1 (> 10)	< 1 (> 100)
GT-45	50 (≥10)	49 (≥ 10)	< 1 (> 10)	< 1 (> 100)
GT-46	49 (≥ 10)	49 (≥ 10)	2 (> 10)	39 (> 100)
GT-47	49 (≥ 10)	49 (≥ 10)	14 (> 10)	< 1 (> 100)

Table S4: Results of additional HBV DNA inhibition testing and cytotoxicity for GT-39, GT-46, and GT-47.

Comp.	Anti-HBV activity in HepAD38 cells	MTS cytotoxicity, IC_{50}, μM
	EC_{50}, μM<br>(% Inh. at 200 μM $\pm$ SD)	IC_{50}, μM<br>(% Inh. at 200 μM)
GT-39	>200 (39.1 $\pm$ 1.5)	>200 (< 1)
GT-46	>200 (25.6 $\pm$ 2.5)	>200 (< 1)
GT-47	>200 (< 1)	>200 (< 1)

Table S5: The range of sampled base and spike angles in deg for all simulated systems. The values are based on the calculated SDEs.

System	Base	Spike	System	Base	Spike
Apo Tetra	31-63	1-45	Apo Hexa	51-71	17-59
GT-32	39-63	3-28	GT-33	43-60	5-28
GT-39	37-61	-1-27	GT-40	43-60	3-30
GT-45	45-63	3-30	GT-46	45-65	6-32
GT-47	44-66	13-40	GLS4	41-57	5-26

Table S6: Fractional area overlaps (FOAs) for SDEs of spike and base angles from MD simulations. Each row shows the FOA of the total SDE for that system with the systems specified in each column. To estimate the differences between the independent simulations for each system, we calculated the overlap area between the SDE of each independent simulation and the SDE from the combined simulations as a fraction of the latter SDE area. Averages of these FOAs from the two independent simulations are shown as diagonal values.

	Tetra	Hexa	GT32	GT33	GT39	GT40	GT45	GT46	GT47	GLS4
Tetra	0.72	0.14	0.43	0.32	0.47	0.39	0.37	0.36	0.32	0.27
Hexa	0.2	0.7	0.15	0.09	0.08	0.11	0.17	0.25	0.41	0.03
GT32	0.91	0.21	0.76	0.66	0.88	0.73	0.76	0.7	0.45	0.56
GT33	1.0	0.19	0.99	0.79	0.97	1.0	0.88	0.85	0.51	0.72
GT39	0.83	0.09	0.75	0.54	0.76	0.61	0.58	0.5	0.29	0.48
GT40	1.0	0.2	0.89	0.81	0.88	0.86	0.8	0.77	0.52	0.61
GT45	0.94	0.29	0.93	0.71	0.83	0.79	0.66	0.85	0.53	0.51
GT46	0.84	0.39	0.78	0.63	0.66	0.7	0.77	0.75	0.68	0.44
GT47	0.68	0.59	0.46	0.35	0.35	0.43	0.45	0.62	0.83	0.23
GLS4	1.0	0.08	1.0	0.84	1.0	0.87	0.74	0.69	0.4	0.91

Table S7: Summarized results of estimated K_D values for selected compounds fitted by the one site specific binding model of Graphpad Prism. The results are listed as the estimated value and the 95% confidence interval.

Compound	K_D (kcal/mol)	95% C.I. (kcal/mol)
GLS4	44.18	31.82 – 63.02
GT-32	123.8	73.14 – 256.9
GT-33	2.862×10^{16}	Minimal
GT-39	7.721×10^{16}	Minimal
GT-40	No binding	N/A
GT-45	7.000×10^{15}	Minimal
GT-46	263.4	156.0 – 615.4
GT-47	3.253×10^{16}	Minimal

Table S8: Summarized results of abnormal capsids fraction for novel compounds. Data is calculated based on randomly selected images of at least 00 capsids. The total number of capsids in the images, the number of abnormal capsids, and the fraction of abnormal capsids are summarized.

Comp.	Trial 1	Trial 2	Average	Standard deviation
	% abnormal capsids (abnormal/total)		%	%
Apo	10 (16/163)	8 (12/152)	9	1
GT-32	76 (92/121)	85 (133/157)	81	4.5
GT-33	31 (38/122)	26 (46/176)	29	2.5
GT-39	45 (52/115)	36 (51/143)	41	4.5
GT-40	8 (11/136)	11 (23/219)	10	1.5
GT-45	40 (48/120)	35 (67/194)	38	2.5
GT-46	70 (97/135)	78 (85/109)	74	4
GT-47	28 (39/141)	23 (37/161)	26	2.5
